# *Neisseria meningitidis* Serogroup W Meningitis Epidemic in Togo, 2016

**DOI:** 10.1093/infdis/jiz330

**Published:** 2019-10-31

**Authors:** Didier Mounkoro, Christelle S Nikiema, Issaka Maman, Souleymane Sakandé, Catherine H Bozio, Haoua Tall, Adodo Yao Sadji, Berthe-Marie Njanpop-Lafourcade, Agoro Sibabe, Dadja E Landoh, Essofa O Abodji, Agbenoko Kodjo, Tsidi A Tamekloe, Téné Alima Essoh, Détèma W Maba, Bradford D Gessner, Jennifer C Moïsi

**Affiliations:** 1 Agence de Médecine Préventive, Dapaong, Togo; 2 Ministère de la Santé et de l’Hygiène Publique, Togo; 3 Institut National d’Hygiène, Lomé, Togo; 4 Agence de Médecine Préventive, Ouagadougou, Burkina Faso; 5 National Center for Immunization and Respiratory Diseases, Centers for Disease Control and Prevention, Atlanta, Georgia; 6 Agence de Médecine Préventive, Paris, France; 7 Agence de Médecine Préventive, Abidjan, Côte d’Ivoire; 8 Organisation Mondiale de la Santé, Bureau Pays, Lomé, Togo

**Keywords:** *Neisseria meningitidis*, epidemic, Togo, vaccination

## Abstract

**Background:**

During 2014, 4 regions in Togo within the African meningitis belt implemented vaccination campaigns with meningococcal serogroup A conjugate vaccine (MACV). From January to July 2016, Togo experienced its first major *Neisseria meningitidis* serogroup W (NmW) outbreak. We describe the epidemiology, response, and management of the outbreak.

**Methods:**

Suspected, probable, and confirmed cases were identified using World Health Organization case definitions. Through case-based surveillance, epidemiologic and laboratory data were collected for each case. Cerebrospinal fluid specimens were analyzed by polymerase chain reaction, culture, or latex agglutination. Vaccination campaigns were conducted in affected districts.

**Results:**

From January 11 to July 5, 2016, 1995 suspected meningitis cases were reported, with 128 deaths. Among them, 479 (24.0%) were confirmed by laboratory testing, and 94 (4.7%) and 1422 (71.3%) remained as probable and suspected cases, respectively. Seven epidemic districts had cumulative attack rates greater than 100 per 100 000 population. Of the confirmed cases, 91.5% were NmW; 39 of 40 available NmW isolates were sequence type-11/clonal complex-11.

**Conclusions:**

This outbreak demonstrates that, although high coverage with MACV has reduced serogroup A outbreaks, large meningococcal meningitis outbreaks due to other serogroups may continue to occur; effective multivalent meningococcal conjugate vaccines could improve meningococcal disease prevention within meningitis belt populations.

Globally, the highest incidence of meningococcal disease is observed in the meningitis belt of sub-Saharan Africa, which extends from Senegal to Ethiopia. Within the belt, seasonal meningitis outbreaks have occurred annually, with large waves every 5–12 years [1, 2]. Historically, such epidemics were caused primarily by *Neisseria meningitidis* serogroup A (NmA) [3]. In 2010, 26 countries in the African meningitis belt began the phased introduction of a meningococcal serogroup A conjugate vaccine ([MACV] MenAfriVac). Since then, the incidence of NmA cases has declined sharply in many countries, including Togo [4–6].

Togo has 3 northern regions that lie within the meningitis belt: Savanes, Kara, and Centrale (Figure 1). In 1997, the first large-scale meningitis epidemic in Togo due to *NmA* was reported, with 2992 cases and a cumulative attack rate of 581 per 100 000 persons in a period of 24 epidemiologic weeks [7]. In 2007, a meningitis epidemic with 723 cases was reported. The causative organisms were *N meningitidis* serogroup W (NmW), *N meningitidis* serogroup X (NmX), and NmA [4–6, 8]. The NmW strain identified in the 2007 outbreak was sequence type (ST)-2881, belonging to clonal complex (CC)-175; this strain was also reported in Benin in the same time period [6].

**Figure 1.**
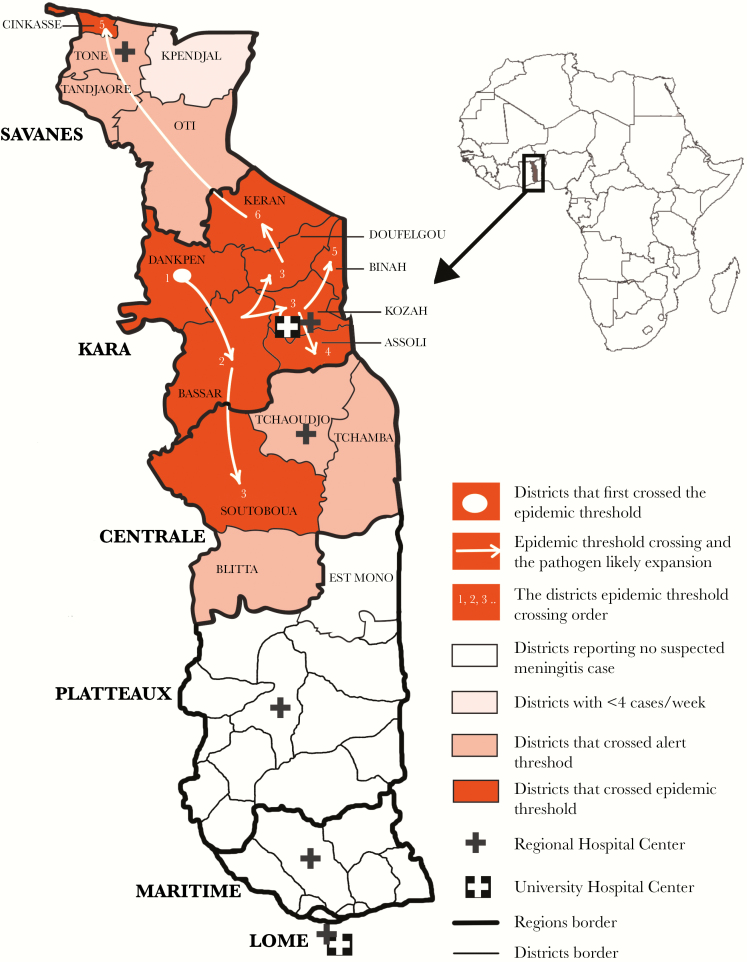
Map of Togo with region including districts that crossed alert and epidemic threshold from January 11 to July 5, 2016 (epidemiologic weeks 2–27).

In the meningitis belt, the main strategies for detecting and controlling epidemics of bacterial meningitis are “enhanced surveillance” based on rapid case detection using a syndromic case definition for bacterial meningitis, treatment of suspected cases with ceftriaxone, and vaccination of persons in affected areas with meningococcal ACYW polysaccharide vaccine. However, polysaccharide vaccines do not provide long-term protection or prevent carriage acquisition [9], and thus they have been unable to prevent recurrent outbreaks. Consequently, in 2014, Togo introduced MACV via a mass vaccination campaign targeting people aged 1–29 years who resided in the 3 regions in the meningitis belt and in the Plateaux region, which borders the Centrale region but is not included in the meningitis belt.

From 1997 until 2016, no large meningitis outbreaks have occurred in Togo based on the number of suspected meningitis cases. However, in 2016, Togo experienced its first large NmW epidemic, which affected 9 districts in the Savanes, Kara, and Centrale regions. This manuscript describes the epidemiology, response, and management of this epidemic.

## METHODS

### Meningitis Surveillance

Togo has 2 complementary meningitis surveillance systems: enhanced surveillance and case-based surveillance. Enhanced surveillance was introduced in Togo in 2003, with the objective of detecting an outbreak by comparing attack rates of suspected meningitis cases to World Health Organization (WHO)-established thresholds to guide an appropriate public health response. Case-based surveillance represents a more intensive approach in which detailed epidemiological data are collected on any individual suspected of having meningitis, and cerebrospinal fluid (CSF) specimens are collected from each patient and analyzed using laboratory testing. In 2014, case-based surveillance was implemented in the Savanes region while other regions continued with enhanced surveillance. The WHO standard meningitis case definitions were used to classify cases [10].

### Laboratory Methods

As part of case-based meningitis surveillance, a lumbar puncture was performed in health facilities on any suspected case of meningitis. The CSF specimen obtained was sent to the nearest peripheral first-level laboratory with a case notification form. The specimens followed a circuit from the first-level laboratory to intermediate regional laboratories, and then to the Institut National d’Hygiène (INH), the national public health reference laboratory for outbreak-prone diseases. Each laboratory performed laboratory analysis corresponding to its technical skills. First-level laboratories performed Gram stain and latex agglutination on CSF specimens. Intermediate-level laboratories performed culture, in addition to repeating the diagnostic testing performed at the first-level laboratories. The INH performed culture and conventional polymerase chain reaction (PCR) [11]. Available isolates and randomly selected CSF specimens were shipped to the WHO Collaborating Center in Oslo, Norway for quality control, testing for antimicrobial resistance testing using the minimum inhibitory concentration method [12] and molecular genotyping [13].

Gram stain results were categorized as Gram-negative diplococci, Gram-positive diplococci, Gram-negative bacilli, or negative. Using Pastorex meningitis Bio-Rad kits, latex agglutination identified NmA, NmC, NmW/Y, NmB/*Escherichia coli* K1, *Streptococcus pneumoniae* (Sp), and *Haemophilus influenzae* type b (Hib). Culture was performed on all CSF specimens received at INH, and PCR assays were performed using gene targets of *crgA*, *lytA*, and *bexA* for Nm, Sp, and Hib, respectively; PCR was also used for serogrouping of *N meningitidis* (Nm) [14].

Molecular typing was used to characterize strains from available NmW isolates. Using multilocus sequence typing, 7 housekeeping genes were sequenced to create an allelic profile called a ST; strains with ≥4 alleles in common were grouped into a CC [15]. Molecular profiles were determined by sequencing *N meningitidis* porinA (PorA) and ferric enterobactin receptor (FetA) genes.

A final case classification was made according to PCR, culture, and latex agglutination test results. Confirmed cases were any suspected or probable cases that had confirmed the presence of Nm, Sp, or Hi in CSF by PCR, culture, or latex agglutination. Probable cases were any suspected cases with the detection of a pathogen by Gram stain. Suspected cases were those that were neither confirmed nor probable.

### Outbreak Response

During epidemiologic week 4, and at the request of the Kara Regional Director of Health, the first investigation was conducted in Dankpen by a team that included staff from Agence de Médecine Préventive (AMP) and the Ministry of Health. During this mission, the causative pathogen of the outbreak was confirmed as NmW. Subsequently, several additional nongovernmental organizations provided support for management of the outbreak. The AMP’s mobile microbiology laboratory was established in Bassar and then in Sotouboua districts to perform Gram stain, latex agglutination, and culture. The mobile laboratory acted as an intermediate laboratory, receiving samples from the first-level laboratories in the Kara and Centrale regions. After laboratory testing, CSF samples and isolates were transported to and stored at INH.

Based on epidemiological and laboratory results, the Togolese Ministry of Health and WHO requested quadrivalent meningococcal polysaccharide vaccines (MPSV4) containing serogroups A, C, Y, and W from the International Coordinating Group (ICG) secretariat to control the epidemic. After the request was approved and vaccines were delivered, vaccination campaigns were conducted in districts that crossed the epidemic threshold (>10 suspected cases per 100 000 population).

Epidemic coordinating committees were set up to coordinate public health interventions through regular meetings, provide updated data, and share information through a situation report. In addition, case management training, treatment protocol updates, and population sensitization on meningitis symptoms were conducted in the field, as needed.

### Statistical Analysis

Incidence rates of suspected meningitis cases were calculated for each epidemiologic week, district, and subdistrict within a district of ≥100 000 inhabitants, using 2016 population estimates from each district and subdistrict as the denominator. Rates at the district level only were compared with WHO-established thresholds to guide the public health response [10]. Vaccine coverage was also estimated by dividing the number of vaccinated people by the target population, using the same population estimates restricted to age groups targeted for vaccination.

All cases recorded in the line-list from January 11 to July 5, 2016, regardless of laboratory confirmation, were included in the analysis. Not all variables were collected for patients from whom CSF samples were collected, and, in these cases, data were recorded as missing. The descriptive analysis was performed using STATA 14 and Microsoft Excel. Maps were created using Adobe Illustrator CC 2015.

Analysis of data collected through routine surveillance and outbreak response was regarded as public health nonresearch, and therefore Institutional Review Board review was not required by any of the participating institutions.

## RESULTS

From January 11 to July 5, 2016, 3 regions (Savanes, Kara, and Centrale) (Figure 1) in Togo experienced a meningitis epidemic with 1995 suspected cases, for a cumulative attack rate of 78.8 suspected cases per 100 000 population (Table 1). Among the 1995 patients with suspected meningitis, 61.3% were aged <15 years, and 51.7% were male. One hundred twenty-eight deaths were reported, and the case fatality ratio was 6.4% (Table 1).

**Table 1.** Attack Rate, Case-Fatality Ratio, and Epidemic Crossing Weeks of Suspected Meningitis Cases by Region, Districts, and Subdistricts Affected by the NmW Epidemic—January 11–July 5, 2016 (Epidemiologic Weeks 2–27) in Togo

REGIONS/Districts/Subdistricts	Population	Cumulative Suspected Cases	Cumulative Attack Rate (per 100 000 Inhabitants)	Epidemic threshold (Suspected Cases/ Week)	Weeks in Which Epidemic Threshold Was Crossed (Number of Suspected Cases)	Cumulative Deaths	Case Fatality Ratio (%)
KARA	881 967	1370	155.3	_	_	82	6.0
Assoli	58 920	117	198.6	6.0	w9 (9); w10 (17); w11 (17); w12 (12); w13 (11); w14 (12); w15 (10); w19 (6)	3	2.6
Bassar	136 513	198	145.0	14.0	w6 (37); w7 (28); w8 (58); w9 (24)	16	8.1
Bassar 1	45 199	_	_	5.0	w5 (7); w6 (12); w7 (5); w8 (19)	_	_
Bassar 2	48 612	_	_	5.0	w8 (13)	_	_
Bassar 3	42 702	_	_	4.0	w6 (13); w7 (12); w8 (15); w9 (13)	_	_
Binah	80 516	116	144.1	8.0	w8 (8); w9 (17); w10 (48); w11 (18)	13	11.2
Dankpen	148 398	354	238.5	15.0	w2 (16); w3 (21); w4 (40); w5 (75); w6 (63); w7 (45); w8 (42); w9 (28)	15	4.2
Dankpen 1	51 263	_	_	5.0	w2 (12); w3 (13); w4 (25); w5 (39); w6 (40); w7 (23); w8 (24); w9 (16)	_	_
Dankpen 2	40 425	_	_	4.0	w3 (4); w5 (12); w6 (8); w7 (10); w8 (7); w9 (5)	_	_
Dankpen 3	56 710	_	_	6.0	w4 (12); w5 (21); w6 (13); w7 (9); w8 (9); w9 (7)	_	_
Doufelgou	90 307	187	207.1	9.0	w7 (15); w8 (34); w9 (16); w10 (17); w11 (26); w12 (22); w13 (19); w14 (9); w16 (14)	13	7.0
Kéran	107 212	188	175.4	11.0	w11 (23); w12 (25); w13 (27); w14 (18); w15 (31); w16 (24); w25 (11)	5	2.7
Kéran 1	43 808	_	_	4.0	w8 (4); w11 (5); w13 (5); w14 (5); w15 (7)	_	_
Kéran 2	36 239	_	_	4.0	w9 (4); w10 (4); w11 (8); w12 (7); w13 (8); w15 (16); w16 (12); w25 (7)	_	_
Kéran 3	27 165	_	_	3.0	w11 (10); w12 (16); w13 (14); w14 (10); w15 (8); w16 (8); w17 (3); w25 (4)	_	_
Kozah	260 101	210	80.7	26.0	w7 (28); w8 (48); w9 (48); w10 (33)	17	8.1
Kozah 1	90 054	_	_	9.0	w11 (11)	_	_
Kozah 2	48 685	_	_	5.0	w8 (9); w9 (13); w10 (10)	_	_
Kozah 3	58 290	_	_	6.0	w8 (12); w9 (9); w10 (12)	_	_
Kozah 4	63 072	_	_	6.0	w7 (13); w8 (12); w9 (16)	_	_
CENTRALE	708 251	239	33.7	_	_	27	11.3
Sotouboua	138 983	113	81.3	14.0	w7 (24); w8 (29); w10 (17)	9	8.0
Sotouboua 1	30 170	_	_	3.0	w5 (4); w6 (5); w7 (5); w10 (3)	_	_
Sotouboua 2	31 643	_	_	3.0	w8 (5); w9 (3)	_	_
Sotouboua 3	26 479	_	_	3.0	w6 (4); w7 (4); w8 (6)	_	_
Sotouboua 4	21 411	_	_	2.0	w8 (2)	_	_
Sotouboua 5	29 280	_	_	3.0	w8 (14); w9 (4); w10 (4)	_	_
SAVANES	941 930	351	37.3	_	_	19	5.4
Cinkansé	89 537	100	111.7	9.0	w8 (9); w9 (13); w10 (19); w11 (25); w12 (10)	2	2.0
Total	2 532 148	1995	78.8	_	_	128	6.4

Regions are indicated in capital character; Districts with more than 100 000 inhabitants are subdivided into subdistricts. Subdistricts are indicated by the districts name + the respective number.

Abbreviations: NmW, *Neisseria meningitidis* serogroup W; w, epidemiologic week.

### Outbreak Detection and Geographic Spread

In epidemiologic week 2 of 2016, the Dankpen district in the Kara region crossed the epidemic threshold, with an attack rate of 10.8 per 100 000 population, and the epidemic continued until epidemiologic week 9 (Figure 1). Additional districts in the Kara region (Bassar, Kozah, Doufelgou, Assoli, Binah, and Keran) crossed the epidemic threshold between epidemiologic weeks 5 and 11. These 7 districts remained in epidemic for 3–8 weeks, with an average duration of 6.3 weeks.

Cases were also detected in districts in other regions, including Sotouboua district in the Centrale region and Cinkassé district in the Savanes region. These districts crossed the epidemic threshold in epidemiologic weeks 7 and 8, respectively, and remained in epidemic for 3–4 weeks. Three districts each in the Savanes and Centrale regions reached the alert threshold but did not cross the epidemic threshold (Figure 1).

**Figure 2.**
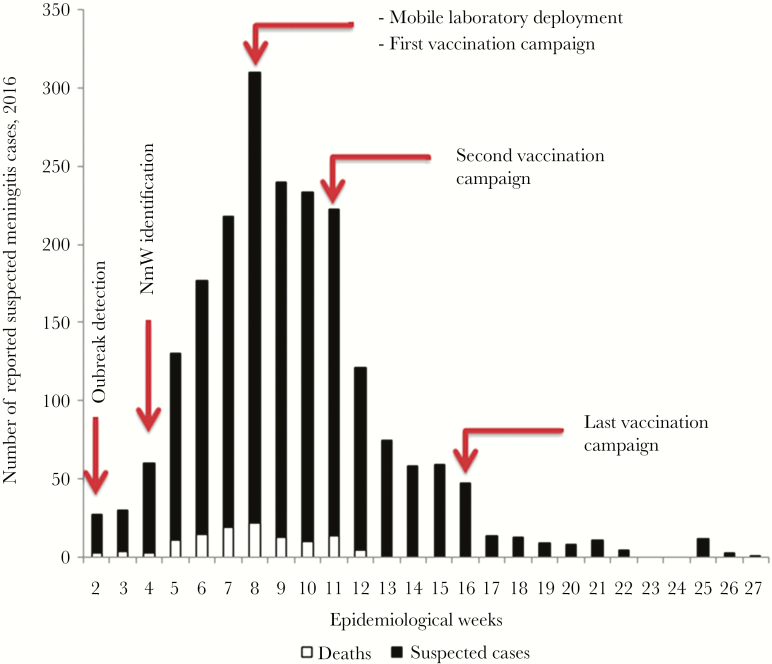
Epidemiologic curve of suspected meningitis cases and deaths by epidemiologic week—January 11–July 5, 2016 in Togo.

During the epidemic, the number of suspected meningitis cases increased rapidly from week 2, peaking in week 8, with over 50 cases per week continuing through week 16, and final cases were reported in week 27 (Figure 2). Among the total of 1995 suspected cases reported, 1370 (68.7%) were from the Kara region. Notably, the cumulative attack rate for this region (155.3 suspected cases per 100 000 population) was 4 times higher than that from the Savanes or Centrale regions (Table 1). Within the Kara region, the Dankpen, Doufelgou, and Bassar districts had the highest cumulative attack rates at 238.5, 207.1, and 198.6 per 100 000 population, respectively (Table 1).

### Laboratory Results

Of the 1995 suspected cases reported, 1366 (68.5%) had CSF specimens collected, 1318 (96.5%) of which had laboratory testing performed. Of tested specimens, 923 (70.0%) were analyzed by PCR, 847 (64.3%) were cultured, 1006 (76.3%) were analyzed by latex agglutination, and 1275 (96.7%) were analyzed by Gram stain. A total of 622 CSF specimens were analyzed by all 4 methods. 

**Table 2.** Age Distribution and Case-Fatality Ratio Among All Suspected Meningitis Cases and Confirmed NmW Cases—January 11–July 5, 2016 (Epidemiologic Weeks 2–27) in Togo

Age Group (Years)	All Suspected Meningitis Cases N (%)	Deaths N (%)	Confirmed NmW Cases N (%)	Deaths Among Confirmed NmW Cases N (%)
<1	270 (13.5%)	8 (3.0%)	41 (9.3%)	1 (2.4%)
1–4	405 (20.3%)	27 (6.7%)	64 (14.5%)	3 (4.7%)
5–9	331 (16.6%)	20 (6.0%)	91 (20.7%)	7 (7.7%)
10–14	214 (10.7%)	10 (4.7%)	68 (15.5%)	2 (2.9%)
15–29	374 (18.7%)	23 (6.1%)	83 (18.9%)	6 (7.2%)
≥30	397 (19.9%)	38 (9.6%)	93 (21.1%)	14 (15.1%)
Missing age	4 (0.2%)	2 (50.0%)	0	0
All ages	1995	128 (6.4%)	440	33 (7.5%)

Abbreviations: NmW, *Neisseria meningitidis* serogroup W.

Based on laboratory results, 479 (24.0%) cases had confirmed bacterial meningitis, and 94 (4.7%) were probable cases; 1422 (71.3%) cases remained suspected cases, including 629 cases that did not have a CSF specimen collected, 48 cases for which CSF was collected but not analyzed, and 745 cases with negative laboratory results for Nm, Hi, and Sp (Table 3). Of the 479 confirmed bacterial meningitis cases, 440 (91.9%) were NmW, 29 (6.1%) were Sp, and 7 (1.5%) were NmX. One NmA case was identified, in an unvaccinated child aged 7 months, by Gram stain and latex agglutination; the CSF specimen was not available for PCR testing. Children aged <15 years accounted for 60% of all confirmed NmW cases (Table 3).

**Table 3.** Laboratory Results According to Gram Stain, Latex, Culture, and PCR January 11–July 5, 2016 (Epidemiologic Weeks 2–27) in Togo

Laboratory Test	Result	Number	Percent
Latex, culture, or PCR	*Neisseria meningitidi*s serogroup W^a^	440	32.2%
	*Neisseria meningitidis* serogroup X	7	0.5%
	*Neisseria meningitidis* serogroup A	1	0.1%
	*Neisseria meningitidis* indeterminate	1	0.1%
	*Streptococcus pneumoniae*	29	2.1%
	*Streptococcus suis*	2	0.1%
	*Haemophilus influenzae*	1	0.1%
	Negative	745	54.5%
Gram stain	Gram-negative diplococci	70	5.1%
	Gram-positive diplococci	16	1.2%
	Gram-negative bacillus	6	0.4%
Not analyzed		48	3.5%
All collected CSF		1366	100.0%

Abbreviations: CSF, cerebrospinal fluid; PCR, polymerase chain reaction.

^a^ *Neisseria meningitidis* serogroup W (NmW) was determined by adding NmW identified by PCR and latex agglutination. Given that latex does not discriminate between the W and Y serogroups, we assumed that the NmW/Y latex results were NmW in the context of this epidemic.

Forty NmW isolates underwent molecular typing and antimicrobial susceptibility testing. All 40 NmW strains belonged to ST-11/CC-11. Furthermore, 39 isolates harbored the PorA 5.2 and FetA 1-1 alleles; the remaining isolate harbored PorA 6.2 and FetA 1–2 alleles. All strains were sensitive to ceftriaxone with a minimum inhibitory concentration of <0.002.

### Vaccination Response

In epidemiologic week 2, the Dankpen district was the first district to cross the epidemic threshold, although laboratory confirmation of NmW was not available until 2 weeks later. Once sufficient laboratory evidence was available to confirm the outbreak etiology, MPSV4 requests were submitted to ICG in epidemiologic week 6 (February 10), week 8 (February 26), and week 12 (March 24); requests could only be submitted for districts that had crossed the epidemic threshold, requiring separate submissions. The requests were approved, and the following quantity of doses was received: 228 100 on February 20, 229 600 on March 12, and 93 500 on April 13, respectively. An additional 5 thousand doses were received from Plan International Togo. Vaccination campaigns were staggered based on the delivery of vaccines. The first campaign was conducted during epidemiologic week 8 in the Dankpen, Bassar, and Sotouboua districts, with the target of vaccinating all persons aged 2–29 years. The second campaign occurred during epidemiologic week 11 in the Kozah, Binah, and Cinkassé districts, targeting persons aged 2–25 years. The last campaign was held during epidemiologic week 16 in the Doufelgou, Kéran, and Assoli districts, also targeting persons aged 2–25 years. The estimated MPSV4 coverage among the target populations was 94% for the Dankpen, Bassar, and Sotouboua districts; 100% for the Doufelgou district; 101% for the Kéran, Binah, and Assoli districts; 102% for the Cinkassé district; and 105% for the Kozah district.

**Figure 3.**
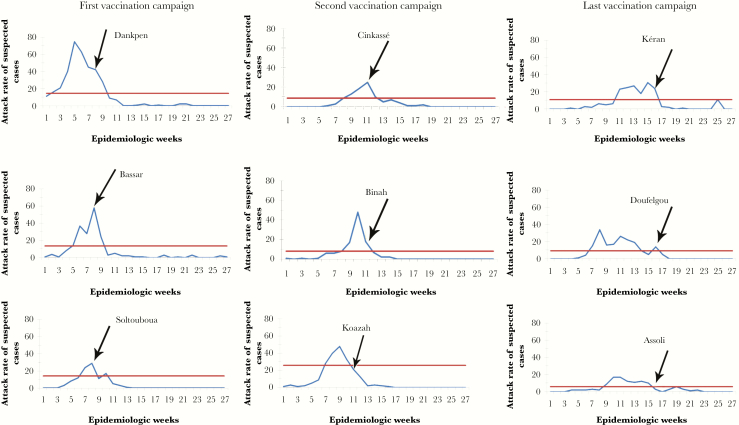
Attack rates of suspected meningitis cases for each district that crossed the epidemic threshold and the beginning of the reactive vaccination campaign—January 11–July 5, 2016 (epidemiologic weeks 2–27) in Togo. The blue line represents suspected cases, the red line represents the epidemic threshold, and the black arrow represents the beginning of vaccination campaign. The epidemic threshold of each district is calculated at its exact value according to its population.

In the Cinkassé, Bassar, and Sotouboua districts, vaccination campaigns were conducted at the peak of the epidemic, and thus it is possible that vaccination altered the upward trajectory in cases (Figure 3). For the remaining 6 districts, vaccination campaigns were conducted after case counts had already begun declining. Three districts experienced an increase in cases at some point after the vaccination campaign.

## DISCUSSION

The 2016, the NmW epidemic in Togo was the third largest epidemic due to this serogroup ever reported in the African meningitis belt [6, 16]. It is the largest NmW epidemic and the second largest epidemic of bacterial meningitis in Togo since 1997 [4, 5, 17, 18]. Among patients with confirmed bacterial meningitis, 91.5% were due to NmW. All of the strains from sequenced isolates belonged to ST-11/CC-11. For 9 districts that crossed the epidemic threshold, the length of epidemic ranged from 3 to 8 weeks. Furthermore, 7 of these districts had a cumulative incidence rate of ≥100 suspected meningitis cases per 100 000 population. A large and well coordinated effort was implemented to respond to the epidemic, culminating with reactive meningococcal polysaccharide vaccination campaigns in 9 districts. Preventive vaccination with a conjugate vaccine containing serogroup W could have more effectively and efficiently prevented the morbidity caused by this epidemic.

Since the introduction of MACV in meningitis belt countries and the near elimination of NmA meningitis, NmW has become one of the predominant causes of meningococcal meningitis in the region [19–22]. However, case counts for NmW epidemics generally have been smaller than for NmA epidemics [5, 16, 19]. The continued occurrence of non-NmA epidemics presents an ongoing risk to the countries of the meningitis belt, because the factors driving these epidemics are still not well understood. There is currently no evidence of capsule switching or serogroup replacement of a niche left by serogroup A [23]. It is plausible that, after the last NmW outbreak in Togo in 2007, population-level immunity waned over time, leading to a higher proportion of susceptible individuals. For example, the 2012 NmW epidemic in Burkina Faso was hypothesized to have occurred 10 years after the 2002 epidemic because of waning population-level immunity [19]. Because Togo experienced an NmW epidemic in 2007, waning population-level immunity may have contributed to the occurrence of the 2016 epidemic.

In addition to this outbreak in Togo, meningococcal meningitis outbreaks due to NmW ST-11/CC-11 have also been reported in Burkina Faso and Niger over the last 15 years [16, 19, 20, 24]. In these outbreaks, more than 75% of NmW cases were in children younger than 15 years of age [19, 24, 25]. However, in this epidemic in Togo, a higher proportion of adults were affected: 21.1% were older than 30 years compared with 3.0% and 4.5% reported, respectively, in Niger in 2015 [24] and Burkina Faso in 2002–2005 [25]. This may have occurred because of relatively low circulation of NmW in Togo over the last decade [5, 26], so that adults have not been exposed and have therefore remained unprotected.

Response activities, targeting different aspects of the outbreak, may have contributed to control of the outbreak. Comparison of attack rates for suspected meningitis cases at the district level with the new WHO epidemic thresholds has improved outbreak detection. The Togolese Ministry of Health, with support from partners, targeted efforts to address staff and antibiotic shortages at the beginning of the epidemic and to expedite diagnostic testing using a mobile laboratory. Despite efforts dedicated to these activities and the vaccination campaigns to control the epidemic, it is unclear to what extent the vaccination campaign altered the course of the epidemic, because vaccination only started at epidemiologic week 8 and, in 5 districts, vaccination campaigns did not start until after the peak in cases. Furthermore, the time between ICG approval for each of the requests and implementing the vaccination campaigns was 2–4 weeks. In addition, for the last 2 vaccination campaigns, the target population was narrowed to specific age groups based on the vaccine quantities received by ICG. Meningococcal vaccination campaigns may have contributed to the decline in disease in some of the districts that had crossed the epidemic threshold, although solid evidence for this is lacking, particularly given the relatively late arrival of vaccine.

Our analysis was subject to some limitations. First, the impact of the vaccination campaign on altering the course of the epidemic is unclear, in part because of the delayed vaccination response, especially relative to peak incidence. Second, the number of confirmed bacterial meningitis cases may have been underestimated, because 34% of all initially suspected meningitis cases did not have CSF specimens collected or whose CSF specimen was not tested. Third, 2.6% of suspected meningitis cases were missing outcome status; consequently, the case-fatality rate could have been underestimated. Fourth, for districts of more than 100 000 inhabitants, the comparison of weekly incidence rates to the established WHO thresholds was done at the district level (not at the subdistrict level), which likely delayed the outbreak detection and subsequent response. For example, the Keran district crossed the threshold at epidemiologic week 11, and its vaccination campaign was held in week 16; however, subdistrict 1 crossed the epidemic threshold at week 8 (3 weeks before the entire district crossed the threshold), so it is possible that a vaccination campaign could have been implemented earlier.

## CONCLUSIONS

Every year, areas in the meningitis belt experience meningococcal meningitis outbreaks, which, since the introduction of MACV via mass vaccination campaigns, have been due to serogroups C, W, or X. The location of outbreaks is unpredictable, due to the culmination of factors including population immunity, pathogen genetic shifts, and a host of individual and environmental risk factors. Consequently, in the absence of a multivalent meningococcal vaccine preventive strategy, affected areas must rely on reactive immunization for outbreak control. However, as this epidemic illustrated, even with motivated staff and a well coordinated response, it may take months to detect, confirm, and mobilize vaccine for an effective reactive vaccination program [27], at which point the outbreak may be waning naturally. Implementation of an effective preventive meningococcal vaccination strategy, combined with continued adherence to strong surveillance with rapid laboratory confirmation to monitor disease trends, identify emerging epidemic strains, guide implementation, and evaluate program effectiveness, could minimize future reliance on reactive vaccination campaigns.

## Notes

***Acknowledgments.*** We thank Ahodo-Abalo Koye and Dr. K. Potchoziou Karabou (Regional Direction of Health, Kara), K. Sylvain Gomez (Regional Direction of Health, Savanes), and Djibril Boere and Cherno Jow (Agence de Médecine Préventive).

The MenAfriNet Consortium (www.menafrinet.org) is an international consortium led and implemented by the (1) Ministries of Health in Burkina Faso, Mali, Niger, Tchad, and Togo, (2) Agence de Médecine Préventive, (3) US Centers for Disease Control and Prevention (CDC), and the World Health Organization (WHO), with support and collaboration from other international and nongovernmental organizations.

***Author contributions.*** D. M., H. T., B.-M. N.-L., A. Y. S., T. A. T., C. S. N., D. E. L., E. A. O., A. S., A. K., and D. W. M. collected surveillance and laboratory data. C. S. N., I. M., D. W. M., A. Y. S., and S. S. cleaned the surveillance data. H. T., B.-M. N.-L., J. C. M., and B. D. G. perform the statistical analysis. D. M., S. S., H. T., C. H. B., and T. A. T. drafted the manuscript. J. C. M. and B. D. G. contributed to study design, overseeing implementation of global meningitis and pneumonia surveillance project, and development of global protocol. T. A. T., B. D. G., and T. A. E. assisted with funding acquisition. All authors contributed in manuscript review.

***Financial support.*** This work was funded by the MenAfriNet consortium (www.menafrinet.org) through a grant from the Bill & Melinda Gates Foundation (OPP1084298).

***Potential conflicts of interest.*** B. D. G. reports the following: grants from Pfizer and the Bill & Melinda Gates Foundation, during the conduct of the study; grants from GSK, Merck, Sanofi Pasteur, Gavi, the Vaccine Alliance, and CDC, outside the submitted work. B. D. G. is currently an employee of Pfizer Vaccines. J. C. M. reports grants from the CDC Foundation, during the conduct of the study. J. C. M. is currently an employee of Pfizer Vaccines. D. E. L. works for the WHO, Togo country office. All authors have submitted the ICMJE Form for Disclosure of Potential Conflicts of Interest. Conflicts that the editors consider relevant to the content of the manuscript have been disclosed.
